# Nutritional Therapies in Congenital Disorders of Glycosylation (CDG)

**DOI:** 10.3390/nu9111222

**Published:** 2017-11-07

**Authors:** Peter Witters, David Cassiman, Eva Morava

**Affiliations:** 1 Metabolic Center, University Hospitals Leuven, B-3000 Leuven, Belgium; emoravakozicz@tulane.edu; 2 Department of Development and Regeneration, Faculty of Medicine, KU Leuven, B-3000 Leuven, Belgium; 3 Department of Gastroenterology-Hepatology and Metabolic Center, University Hospitals Leuven, B-3000 Leuven, Belgium; david.cassiman@kuleuven.be; 4 Hayward Genetics Center, Tulane University School of Medicine, New Orleans, LA 70112, USA

**Keywords:** galactose, mannose, congenital disorders of glycosylation (CDG), treatment, glycosylation, diet

## Abstract

Congenital disorders of glycosylation (CDG) are a group of more than 130 inborn errors of metabolism affecting *N*-linked, *O*-linked protein and lipid-linked glycosylation. The phenotype in CDG patients includes frequent liver involvement, especially the disorders belonging to the *N*-linked protein glycosylation group. There are only a few treatable CDG. Mannose-Phosphate Isomerase (MPI)-CDG was the first treatable CDG by high dose mannose supplements. Recently, with the successful use of d-galactose in Phosphoglucomutase 1 (PGM1)-CDG, other CDG types have been trialed on galactose and with an increasing number of potential nutritional therapies. Current mini review focuses on therapies in glycosylation disorders affecting liver function and dietary intervention in general in *N*-linked glycosylation disorders. We also emphasize now the importance of early screening for CDG in patients with mild hepatopathy but also in cholestasis.

## 1. Introduction

Congenital disorders of glycosylation (CDG) are a family of diseases with the common denominator that they all affect the most important posttranslational modification of proteins, i.e., glycosylation [1]. They were initially described by Jaak Jaeken and currently compromise a group of more than 130 separate entities [1]. The most abundant type of CDG affect *N*-linked glycosylation. Most of these CDG are multisystemic diseases with hepatic involvement [2].

*N*-glycosylation is initiated with the activation of sugars in the cytoplasm of the cell. These phosphorylated sugars are subsequently transferred to the lipid dolichol, in the membrane of the endoplasmic reticulum (ER). This stepwise enzymatic assembly process leads to the formation of a glycan molecule. The base of this glycan consists of *N*-acetyl glucosamines (GlcNac), followed by several mannose (Man) residues. It is first assembled at the cytoplasmic surface and later inside the ER. Finally, the addition of glucose molecules is a signal to release the glycan from dolichol, followed by the attachment of the glycan to a protein. In the case of *N*-glycosylation, the glycan is linked to an amino group of an asparagine (N). The final steps of the glycosylation process take place in the Golgi apparatus with tailoring of the glycan molecule to its mature form by trimming away some of the mannose units and adding galactose and sialic acid [3,4].

While the assembly process in the ER is almost fully understood because it is conserved down to yeast and the consecutive building steps mirror each other, the process in the Golgi is very complicated as the success of the end product depends on several other factors outside the classic glycosylation steps. This glycosylation relies on the proper function of several transporters making the activated sugars available for synthesis. Defects in Golgi proteins that affect the trafficking and the transport of glycoproteins throughout the Golgi (functioning for example in the transport of protons and trace elements) can also lead to hypoglycosylation [3]. This is a rapidly growing family of newly described CDG [1,5].

The diagnosis of *N*-linked glycosylation disorders is made by serum transferrin isoelectric focusing, or mass spectrometry of transferrin isoforms. Depending on whether the defect is localized in the cytoplasm or endoplasmic reticulum (E.R.) or in the Golgi compartment, a different pattern can be observed, (type I and type II, respectively) [6].

In *O*-linked glycosylation, the glycan is linked to the OH-group of serine or threonine. *O*-linked glycan defects are often tissue specific. The mucin type of *O*-glycosylation is Golgi related and frequently associated with *N*-linked glycosylation defects. It can be screened by isoelectric focusing of serum apolipoprotein C-III, that is only *O*-glycosylated [3]. A well-known group of tissue specific *O*-glycosylation defects are the dystroglycanopathies [1,7].

Glycosylphosphatidylinositol (GPI)-anchored glycosylation defects is a growing group of genetic disorders clinically characterized by intellectual disability and biochemically by hyperphosphatasia. GPI anchors attach a number of proteins, including alkaline phosphatase, to membranes. Hyperphopsphatasia reflects the inability of the ectoenzyme (alkaline phosphatase) to anchor to the membrane and hence this will continue to circulate causing the hyperphosphatasia [3]. Lipid linked glycosylation is also important for the correct anchoring of T-cell antigens. The best known GPI-anchor defect, paroxysmal nocturnal hemoglobinuria, is a somatic disorder [4,7].

In CDG there is an important role of the dolichol phosphate mutase (DPM) complex. This complex is involved in the activation of mannose. Activated mannose is needed in all three glycosylation pathways *N*-linked, *O*-linked, and GPI-anchored [3]. The DPM complex has three subunits (DPM1, DPM2 and DPM3). Genetic defects in the DPM complex result in severe multisystem phenotypes, with biochemical anomalies of all three pathways (combined glycosylation defects) [3]. The phenotype consists of typical *N*-glycosylation symptoms, such as seizures, microcephaly, strabismus and developmental disability, but also eye anomalies and brain migration defects, seen in the dystroglycanopathies [8].

Liver disease is a feature of nearly all *N*-linked CDG. It mostly involves increased levels of transaminases (for example in the most prevalent CDG, PMM2-CDG). But sometimes it can manifest as hepatomegaly, cholestasis or liver failure. Histologically, liver fibrosis, ductal plate malformation, cirrhosis and steatosis have been observed. Especially the recently described CDG subtypes involving the Golgi can have liver disease, as their predominant feature (ATP6AP1-CDG, TMEM199-CDG and CCDC1115-CDG) [2,9,10,11]. In several *N*-linked CDG, liver transplantation has been necessary, due to progressive cirrhosis, for instance in MPI-CDG, or CCDC1115-CDG. Not only is transferrin, central to the diagnosis of *N*-linked CDG, produced by the liver, but so are a myriad of secreted and glycosylated proteins. For example, coagulation factors, except for factor VIII, are solely produced by the liver. In many CDG, there are disturbances of the coagulation profile. Usually this is due to a synthesis defect with decreased levels of factor IX, XI, protein C, antithrombin III and protein S, and also decreased glycosylation of these factors [12].

Dietary therapy is an important intervention in CDG [13]. Only a few CGD subtypes are “treatable”. This article focuses on the different treatment options in *N*-linked glycosylation disorders with liver involvement. 

## 2. Materials and Methods

The present literature review started with the search of the Medline database, using PubMed as search engine (last accessed October 2017). AND and OR operators were used in the database search to combine the keywords “therapy”, “treatment”, “supplement” or “diet” in combination with CDG or the two common full names for CDG: (a) carbohydrate-deficient glycoprotein syndrome(s) and (b) congenital disorder(s) of glycosylation. For the search algorithm see Supplementary File S1. Papers were included in present review if they contained information on potential nutritional treatments in CDG that are known to affect the liver. Additionally, clinical trials were searched on www.ClincalTrials.gov using the same keywords.

## 3. Results

Two hundreds and forty-three articles matching these search terms were identified. Selection of the articles on *N*-glycosylation and describing nutritional therapy in at least 1 patient or a relevant animal model. Forty-nine articles were selected for further study. This information was complemented with the information presented at the World CDG-conference 2017 and the registered trials on Clinicaltrials.gov.

### 3.1. Specific Dietary Therapies in Selected CDG Types

Several CDG were found to be (at least partially) treatable by nutritional interventions such as mannose, galactose, etc. (Table 1).

### 3.2. Mannose in PMM2-CDG (MIM # 212065)

PMM2-CDG is the first described CDG and is due to phosphomannomutase-2 (PMM2) deficiency. It has two clinical presentations. The neurological subtype presents with cerebellar atrophy with ataxia, intellectual disability, seizures, retinopathy, stroke like episodes and peripheral neuropathy [14]. The more severe and potentially lethal multisystem phenotype, also has organ involvement such as hepato-gastrointestinal (chronic diarrhea, protein-losing enteropathy, liver failure, cirrhosis), cardiac and kidney disease [15]. Liver involvement (mainly hepatomegaly and raised transaminases) is present in up to 50% of patients [2].

Nutritional therapy has been considered and trialed in the past in these patients. In vitro, there was a clear improvement observed in the glycosylation pattern by treating patient fibroblast lines with mannose [16,17]. However, clinically, based on multiple clinical reports (but no structural therapy trials), there has been no confirmed clinical improvement, nor biochemical improvement at the level of the defective glycosylation, in patients [17,18].

As of now we have to conclude that mannose therapy is not effective in PMM2-CDG. Evaluating the reported laboratory data in PMM2 deficient patients is also difficult, since in older patients, transferrin isoforms may normalize spontaneously, and in many patients with PMM2-CDG there is a gradual improvement of transaminases over time [15], parallel with clinical stabilization. There is an absolute need for future nutritional therapeutic trials; ideally double blind randomized controlled trials, in PMM2-CDG.

### 3.3. Mannose in MPI-CDG (MIM # 602579)

MPI-CDG is longest known treatable CDG-type. It is characterized by bleeding diathesis, increased risk for thrombotic events, abnormal liver function, hyperinsulinism and chronic diarrhea. Patients have a normal intellect. Transferrin analysis shows a type I pattern. On liver histology congenital hepatic fibrosis, microvesicular steatosis and fibrosis or cirrhosis has been documented.

Initial investigations showed successful restoration of glycosylation by mannose in vitro which was followed by compassionate use of mannose in MPI-CDG patients [19]. This is not surprising because mannose can be phosphorylated by the hexokinase so that the defective phophomannose isomerase (MPI) that converts fructose-6-phosphate to mannose-6-phosphate can be bypassed. The dose of 200 mg/kg 4–6 times/day was suggested to keep serum mannose levels high enough to alleviate endocrine abnormalities, coagulation defects and the chronic protein-losing enteropathy [19,20,21]. In some of the patients, a higher dosage was needed for full recovery. In a low percentage of patients high doses of mannose led to hemolysis and jaundice. It can also lead to seizures [22]. Coagulation abnormalities and hyperinsulinism usually improve within a few weeks of mannose supplements, both on intravenous and on oral therapy. Chronic diarrhea may recur in exceptional cases. Mannose therapy does not prevent further hepatic injury and about 1/3 of the patients develop liver cirrhosis, sometimes requiring liver transplantation [21,23]. Formal placebo-controlled clinical studies of oral or IV mannose in MPI-CDG patients have not been performed so far, so the data is anecdotal.

### 3.4. Galactose in PGM1-CDG (MIM # 614921)

PGM1-CDG is a combined disorder of glycogenolysis, glycolysis and glycosylation. Most patients are born with a midline defect of the palate, cardiomyopathy and multiple laboratory abnormalities including abnormal coagulation, endocrine parameters and liver function tests. Hypoglycemia is partially due to hyperinsulinism and the abnormal glycogenolysis in patients. Short stature is associated with feeding difficulties. Patients have normal intellect [24].

Phosphoglucomutase 1 (PGM1) deficiency is a unique glycosylation disorder showing a mixed type of glycosylation defect (type I/II). The most characteristic finding is decreased galactosylation additional to a global decrease in glycan synthesis. Based on the decreased number of galactose molecules in truncated glycans and the success of galactose supplements in vitro in patient fibroblasts, recently clinical trials were initiated to evaluate the success of galactose treatment in PGM1 patients [24]. Pilot studies showed an improvement in liver transaminases, coagulation factors (antithrombin III and factor XI) and in a variable degree of endocrine parameters. The frequency of rhabdomyolysis decreased, however, the treatment did not affect muscle weakness and creatine kinase (CK) levels [25,26]. A frequent complex carbohydrate rich diet remains necessary to keep blood glucose levels in the normal range in all patients. In three patients, transient tube feeding had been needed due to severe recurrent hypoglycemic episodes. The frequency of hypoglycemic episodes however did improve on d-galactose therapy.

### 3.5. Galactose in SLC35A2-CDG (UDP-Galactose Transporter) (MIM # 300896)

This CDG is due to a deficiency of the UDP-galactose transporter (solute carrier (SLC) 35A2) in the Golgi leading to a type II pattern on transferrin analysis. Clinically it manifests as an early infantile epilepsy with developmental delay, hypotonia, variable ocular anomalies, and brain malformations (cerebellar atrophy, delayed myelination and a thin corpus callosum) [27]. Many of the patients have a mosaic form of the mutation, not showing transferrin abnormalities. Non-mosaic patients have significant elevation of the transaminases.

In one patient, glycosylation could be nearly completely restored by galactose supplementation [28]. Although seizures tend to improve on galactose therapy, transaminases remain elevated (personal communication, European Metabolic Group conference 2016).

### 3.6. Galactose in SLC39A8-CDG (Manganese transporter) (MIM # 616721)

This type II CDG is a recently described multi-systemic neurodevelopmental disorder with phenotypes ranging from cranial synostosis, hypsarrhythmia and disproportionate dwarfism to strabismus, cerebellar atrophy, hypotonia, intellectual disability and recurrent infections [1]. Liver transaminases can be chronically mildly elevated. SLC39A8 is required for the manganese homeostasis in the Golgi, where manganese is a cofactor of the β-1,4-Galactosyltransferase. Unsurprisingly, manganese supplementation [29] and galactose supplementation [30,31] have been attempted. Galactose generally improves glycosylation and high dose manganese treats the epilepsy.

### 3.7. Galactose in TMEM165-CDG (MIM # 614727)

TMEM165-CDG, a type II CDG, combines impaired *N* and *O*-glycosylation and manifests with striking osseous changes as epiphyseal, metaphyseal, and diaphyseal dysplasia. Other features that accompany this peculiar skeletal phenotype are muscular hypotrophy, fat excess, partial growth hormone deficiency, and, in some patients, episodes of unexplained fever. Biochemically there is a mild to moderate increases of serum transaminases (particularly of aspartate transaminase (AST)), CK, and lactate dehydrogenase (LDH), as well as decreased coagulation factors VIII, IX, XI, and protein C [32,33].

Galactose therapy has very recently shown to be beneficial in patient fibroblasts improved glycosylation as well as biochemical parameters (blood coagulation) [5]. This could be due to an upregulation of manganese dependent transferases in the Golgi, such as B4GALT1 (β-1,4-Galactosyltransferase 1).

### 3.8. General Symptom Directed Dietary Therapy in CDG

Next to the specific treatment of several *N*-linked CDG, there are also nutritional treatments of certain syndromes that are often present in CDG.

For instance, hyperinsulism is a well-known feature of MPI-CDG, PGM1-CDG and PMM2-CDG. This can be treated by dietary interventions, such as using foods with a low glycemic index, nocturnal tube feeding and the use of uncooked corn-starch in children above 1 year of age [25]. Most patients need additional diazoxide therapy [25].

There is often a component of failure to thrive in CDG patients. In these patients complimentary feeding and tube feeding can be necessary. Moreover, an elementary diet can prove to be useful.

Protein losing enteropathy is a main feature of MPI-CDG but can also be present in the most prevalent CDG (PMM2-CDG). In some patients lymphangiectasia have been documented and a MCT diet has shown to be useful [12,34]. Somatostatin treatment is an additional non-nutritional intervention.

MAN1B1-CDG is the only known CDG (type II) that is known to be associated with obesity in addition to slight facial dysmorphism and psychomotor retardation [35]. In this CDG, caloric restriction can be necessary.

Similar to MELAS (mitochondrial encephalomyopathy, lactic acidosis, and stroke-like episodes) syndrome, l-arginine has been used to treat stroke-like episodes in CDG. l-arginine could improve NO production and hence vasodilation in these patients (personal communications, Prof P de Lonlay). However, no formal trials have been performed.

In CDG presenting with cholestasis (such as ALG8-CDG, COG6-CDG, COG7-CDG, CCDC115-CDG and ATP6AP1-CDG), nutritional interventions such as in other causes of cholestasis can be necessary [2]. These entail the supplementation of fat-soluble vitamins and MCT (medium-chain triglycerides) that are more easily absorbed in the absence of bile [36].

In pediatric neurology, the ketogenic diet has proven to be useful in controlling refractory seizures [37]. In CDG refractory seizure are rare, but the ketogenic diet could also be used. One issue complicating this is the occurrence of hyperinsulinism in some CDG often leading to hypoglycemia [15] while on the ketogenic diet as this contains a low amount of carbohydrates [37].

Dietary treatment of CDG has been mostly successful with the traditional method (fructose and lactose restrictions), however no clinical trial is available about the efficacy of this diet. Alcohol use worsens the liver phenotype in CDG significantly, and oppresses lipid linked oligosaccharide synthesis. The alcohol-related glycosylation abnormality is reversible.

## 4. Discussion

Congenital Disorders of Glycosylation are usually multisystem disorders, mostly affecting the central nervous system, in addition to diverse laboratory anomalies. *N*-linked glycosylation defects are always affecting liver function, leading to abnormal synthesis of secretory proteins, including albumin, hormone transporters, and coagulation and anticoagulation factors, partially due to abnormal synthetic function of the liver, but also due to a disturbed glycosylation and insufficient posttranslational regulation of important functional proteins [12]. Alpha 1 antitrypsin, ceruloplasmine and other liver proteins are frequently abnormal. Liver transaminase levels are almost always elevated, although spontaneous recovery is common. Hepatomegaly is uncommon and only a few *N*-linked disorders lead to cholestasis, fibrosis or cirrhosis [2,9].

Jaundice is rarely present. In fact, only two recently described disorders (CCDC115-CDG and ATP6VAP1-CDG) are cholestatic conditions. In addition, some patients had to undergo liver transplantation [9,10].

This is also the case for MPI-CDG, where a cryptogenic liver disease can progress to liver cirrhosis without overt symptoms, even on successful dietary therapy.

The biochemical background of dietary monosaccharide therapy in CDG is not completely understood. Mannose therapy has been for long the only treatment in Mannose-Phosphate Isomerase (MPI) deficiency. The background of the therapy is that the enzyme block in MPI-CDG does not allow producing sufficient amount of mannose-6-phosphate for the endoplasmic reticulum (ER) related glycosylation. Using a very high concentration of mannose, administered every 4 hours, could salvage this [20]. By this concentration mannose can be directly activated (phosphorylated) by hexokinase. This phosphorylation step requires a higher than physiologic concentration of mannose since the enzyme’s natural substrate is glucose, and hexokinase is only moonlighting in this reaction under these conditions. Mannose supplements unfortunately do not solve the clinical problem in PMM2 deficiency where a lack of mannose-1-phosphate makes further mannose activation impossible [38]. Experimental treatments in fibroblasts using chemically disguised mannose-1-phosphate are ongoing [13].

The theory behind oral galactose treatment is different. In SLC35A2-CDG the galactose transporter appears to be upregulated by excess of galactose, which leads to an increase in galactose transport to the Golgi in vitro [28]. This mechanism is similar to that seen by defects of the fucose transporter SLC35A1 (SLC35A1-CDG), which also shows an improved function on oral fucose therapy, with improving glycosylation. In both cases, transferrin glycosylation increases significantly but also some of the clinical features improve [13].

Galactose mode of action is different in PGM1-CDG. The hypothesis is that increasing galactose concentrations in blood increase galactose-1-phosphate and UDP-galactose levels, which restore the altered balance within abnormal nucleotide sugar pools. This would improve hypogalactosylation in the Golgi. Interestingly, in some unknown way, galactose supports the ER related glycosylation as well in PGM1 deficiency, where it has been shown that lipid linked oligosaccharide synthesis is arrested, but recovers on in vitro galactose treatment [24,25,26]. Galactose might offer extra energy substrate for patients, since some of the adults with PGM1 deficiency also show an improvement of their muscle disease [39].

In TMEM165, galactose’s positive effect is suspected secondary through B4GALT1, a Golgi enzyme, which is both manganese and galactose concentration sensitive. TMEM165 defect leads to abnormal manganese transport to the Golgi, affecting Golgi oligotransferases, which apparently improve their function with extra galactose, and increase galactosylation [5]. This is similar to galactose effect in SCLA39A8-CDG, providing manganese for the Golgi, and also for B4GALT1, where both manganese and galactose therapy restores glycosylation, however a cautiously administered high dose of oral manganese therapy is clinically more efficient [29].

Dietary intervention is an evolving and increasingly used therapy in CDG. More and more subtypes are trialed on monosaccharide supplementation, due to the relatively high safety, especially compared to experimental drug trials, and the ease of supplementation. Monosaccharides can be mixed with any type of food and usually have a pleasant, if any taste. Previous experience with monosaccharide therapy in individual cases showed that increasing the concentration of specific monosaccharides can lead to an increase in Golgi availability. This has been demonstrated by improvement in galactosylation on galactose therapy in SLC35A2-CDG [27,28]. Similar results were observed on fucose therapy in fucose transporter deficiency [40]. Based on these observations one should hypothesize a potential beneficial effect of sialic acid in SLC35A1-CDG, or of *N*-acetyl Glucosamine (GlcNAc) in SLC35A3-CDG as GlcNAc has been already used in the past safely in patients with different medical conditions, for example as supportive therapy in chronic osteoarthritis, as a health supplement. Oral sialic acid supplementation, however has not yet been proven to be efficient and its long acting form was recently withdrawn from clinical trials, due to not reaching any of the study end-points in GNE-CDG) [13]. The problem with the dietary use of sialic acid is that this molecule is not efficiently taken up by the cell and in the Golgi compartment as an oral therapy.

Additional dietary therapies could aim at increasing UDP concentrations in the cell, to have sufficient UDP available for the synthesis of UDP-sugars [24]. This novel approach has been successfully used in CAD-CDG [41]. This has not been systematically trialed in other CDGs. Other possible therapies include manganese supplementation in CDGs related to defective manganese transport, already shown to be effective in two cases of SLC39A8-CDG [29], and manganese can be probably a potential adjuvant therapy for TMEM165-CDG [5].

The question is, whether these dietary interventions are sufficient, and potentially the most efficient interventions in CDG? We learnt from the lesson on MPI-CDG, that mannose therapy is not only risky, when applied in higher doses, but also cannot prevent the progressive fibrotic liver disease in about one third of the patients [23]. Galactose therapy has beneficial effects in several CDGs but does not fully alleviate all clinical symptoms. The long term future of CDG therapy is most likely the use of activated monosaccharides instead of using single dietary sugars. In order to increase substrate concentrations, or supplement missing substrates, we should administer a more specific therapy by ingesting only a small amount of the active compound, compared to the currently used large amounts of simple sugars (in some cases 50 g/monosaccharide added to the diet daily). The efficacy of these novel potential drugs should be however carefully evaluated, for toxicity. The use of animal models is imperative instead of using cell culture models, since a successful cellular delivery of monosaccharides in fibroblasts does not mean that the oral supplementation would be effective [18,38].

## 5. Conclusions

In summary, in our current mini review we evaluated the different nutritional therapy options, associated with a positive effect on liver function in CDG. We believe this new type of nutritional therapy holds great promise for the future and over the last decade numerous CDG have been transformed to at least partially treatable disorders.

## Figures and Tables

**Table 1:** Specific dietary treatments in *N*-linked disorders of glycosylation.

	PMM2-CDG	MPI-CDG	PGM1-CDG	SLC 35A2-CDG	SLC39A8-CDG	TMEM165-CDG
Mannose	**-**	**X**	**-**	**-**	**-**	**-**
Galactose	**-**	**-**	**X**	**X**	**X**	**X**
Frequent complex carbohydrate feeding	**+/−**	**+/−**	**X**	**-**	**-**	**-**
Manganese	**-**	**-**	**-**	**-**	**X**	? *

Abbreviations: CDG: congenital disorders of glycosylation, PMM2: phosphomannomutase 2; MPI: Mannosephosphate Isomerase. * Manganese only trialed in vitro, cautious use is potentially useful as oral therapy. X Clinically trialed dietary supplement with positive biochemical and clinical effects. +/− This therapy has shown beneficiary effect in some of the patients.

## Supplementary Material

The following are available online at www.mdpi.com/2072-6643/9/11/1222/s1, File S1: Search algorithm used in Pubmed (https://www.ncbi.nlm.nih.gov/pubmed/), accessed on 1 October 2017.

## Abbreviations

CDG	Congenital disorders of glycosylation
ER	Endoplasmic reticulum
GlcNac	*N*-acetyl glucosamines
Man	Mannose
GPI	Glycosylphosphatidylinositol
DPM	Dolichol phosphate mutase
PMM2	Phosphomannomutase-2
MPI	Phophomannose isomerase
PGM1	Phosphoglucomutase 1
CK	Creatine kinase
SLC	Solute carrier
AST	Aspartate transaminase
LDH	Lactate dehydrogenase
MELAS	Mitochondrial encephalomyopathy, lactic acidosis, and stroke-like episodes
