# Supplementary material for: Nutritional Therapies in Congenital Disorders of Glycosylation (CDG)
Source: Nutrients. 2017 Nov 7;9(11):1222. doi: 10.3390/nu9111222 (PMC5707694; doi:10.3390/nu9111222)
Supplement: Supplementary file 1 [file nutrients-09-01222-s001.pdf]

File S1: Search algorithm used in Pubmed (https://www.ncbi.nlm.nih.gov/pubmed/), accessed on 1 October 2017).

"dietary supplements"[MeSH Terms] OR ("dietary"[All Fields] AND "supplements"[All Fields]) OR "dietary supplements"[All Fields] OR "supplement"[All Fields]) OR ("diet"[MeSH Terms] OR "diet"[All Fields]) OR ("nutritional status"[MeSH Terms] OR ("nutritional"[All Fields] AND "status"[All Fields]) OR "nutritional status"[All Fields] OR "nutrition"[All Fields] OR "nutritional sciences"[MeSH Terms] OR ("nutritional"[All Fields] AND "sciences"[All Fields]) OR "nutritional sciences"[All Fields]) OR ("therapy"[Subheading] OR "therapy"[All Fields] OR "therapeutics"[MeSH Terms] OR "therapeutics"[All Fields]) AND ("congenital, hereditary, and neonatal diseases and abnormalities"[MeSH Terms] OR ("congenital"[All Fields] AND "hereditary"[All Fields] AND "neonatal"[All Fields] AND "diseases"[All Fields] AND "abnormalities"[All Fields]) OR ("congenital"[All Fields] AND "disorder"[All Fields]) OR "congenital disorder"[All Fields]) AND s[All Fields] AND ("glycosylation"[MeSH Terms] OR "glycosylation"[All Fields]) AND "humans"[MeSH Terms]
